# Realistic artificial DNA sequences as negative controls for computational genomics

**DOI:** 10.1093/nar/gku356

**Published:** 2014-05-06

**Authors:** Juan Caballero, Arian F. A. Smit, Leroy Hood, Gustavo Glusman

**Affiliations:** Institute for Systems Biology, 401 Terry Ave. N, Seattle, WA 98109, USA

## Abstract

A common practice in computational genomic analysis is to use a set of ‘background’ sequences as negative controls for evaluating the false-positive rates of prediction tools, such as gene identification programs and algorithms for detection of *cis*-regulatory elements. Such ‘background’ sequences are generally taken from regions of the genome presumed to be intergenic, or generated synthetically by ‘shuffling’ real sequences. This last method can lead to underestimation of false-positive rates. We developed a new method for generating artificial sequences that are modeled after real intergenic sequences in terms of composition, complexity and interspersed repeat content. These artificial sequences can serve as an inexhaustible source of high-quality negative controls. We used artificial sequences to evaluate the false-positive rates of a set of programs for detecting interspersed repeats, *ab initio* prediction of coding genes, transcribed regions and non-coding genes. We found that RepeatMasker is more accurate than PClouds, Augustus has the lowest false-positive rate of the coding gene prediction programs tested, and Infernal has a low false-positive rate for non-coding gene detection. A web service, source code and the models for human and many other species are freely available at http://repeatmasker.org/garlic/.

## INTRODUCTION

Genomes evolve by random accumulation of mutations and by selection for a variety of functional requirements. For species with short generation time and large population sizes (e.g. bacteria), the strong selective forces lead to highly optimized genomes, dense in genes and with negligible overhead of non-functional sequences: this makes prokaryotic gene prediction relatively straightforward (1). In contrast, the genomes of species with much longer generation times and much reduced population sizes (e.g. vertebrates) accumulate vast amounts of genetic material that largely appears not to be under selective constraints (2). Over half the human genome is derived from retrotransposed elements, DNA transposons, and other types of repetitive sequences (3). Functional sequences and regulatory elements are a small fraction of the vertebrate genome, making their identification difficult. Most vertebrate genes are interrupted by introns replete with material that is largely under low selective constraints; long introns are particularly difficult to model. Recognizing alternative splicing demands further algorithmic complexity, as does modeling of non-coding transcripts. For all these reasons and more, *ab initio* vertebrate gene prediction poses a significant challenge for computational biology.

Current sequencing technologies make it possible to sequence a complete genome in a short time. The next-generation technologies will soon enable the sequencing of a human genome for $1000 in less than a day (4,5). Many other genomes have been sequenced and have high-quality assemblies, including fruit fly (6), mouse (7), chicken (8), chimp (9), dog (10), pig (11), cat (12), horse (13), cow (14) and zebrafish (The Danio rerio Sequencing Project, http://www.sanger.ac.uk/Projects/D_rerio/). With the exponential increase in genome sequences, robust annotation systems are needed to identify all functional elements in a comprehensive and efficient manner.

One of the first analytical steps after a genome is sequenced and assembled is to identify all repetitive sequences, both those derived from the propagation of repetitive elements such as transposons, and ‘tandem repeats’ that arise by expansion of a few nucleotides. RepeatMasker (http://www.repeatmasker.org/) is a common method for identification of repetitive sequence derived from transposable elements. Tools such as the Tandem Repeat Finder (TRF) (15) are used to identify low complexity sequence expansions in the genome. Repetitive sequence detection is challenging because many of the repeats have evolved over millions of years, accumulating substitutions, insertions and deletions to the point of being nearly indistinguishable from random sequence. This problem calls for the development of standard negative and positive controls to evaluate the accuracy of any repeat finder.

The next step in genome analysis is the identification of genes. Coding genes are the best understood functional part of the genome and many tools have been developed to identify them from the genomic DNA sequence. In parallel, non-coding genes can also be detected using various strategies. Modern gene prediction programs rely on three basic concepts:

1. *Modeling of gene structure.* Typical *ab initio* programs look for known components of a gene, such as promoter elements, splicing signals, open reading frames (ORFs) and codon usage (in the case of coding genes) or special folding structures (for non-coding RNAs). Examples of coding gene prediction programs are Genscan (16), Twinscan (17) and Augustus (18).
2. *Similarity-based identification.* Genes can be identified or inferred by local alignment to databases of expressed sequence tags (ESTs), cDNAs, known mRNAs or protein sequences. The expression data can be derived from the same organism or from related model organisms, which may have more detailed annotation. Sequences may thus inherit a classification or function based on their similarity to a reference sequence. Examples of programs in this category are N-Scan (19) and JIGSAW (20). Specialized programs exist for the detection of specific RNA families such as SnoScan (21) and tRNAscan (22), while Infernal (23) can use diverse models for detection of ncRNAs.
3. *Detection of transcriptional footprints.* Observing various signatures of transcription that accumulated over evolutionary time can identify transcribed regions. This includes biased mutation rates (24) and strand-biased representation of interspersed repeats and poly-adenylation signals (25). These two approaches are embodied in the FEAST tool (25), which relies on four algorithms to identify transcribed regions: Greens and CHOWDER quantify mutational strand biases caused by transcription-coupled DNA repair, and ROAST and PASTA are based on strand-specific selection against disruptive poly-adenylation signals.

**Figure 1.**
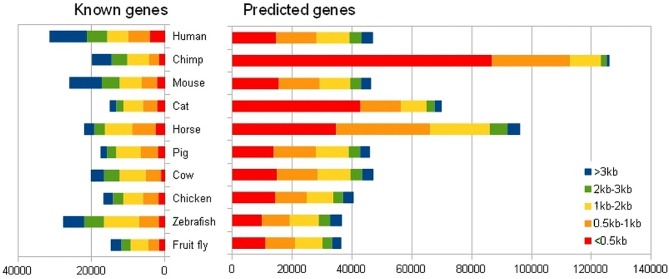
Total number of known and predicted coding genes in Ensembl 64 for human and other species. The gene size is determined by the total length in bases of each CDS.

Most gene prediction programs use a data set of sequences with validated genes for training: each program ‘learns’ what a region containing a gene looks like. Then the program scans a sequence, scoring fragments for gene plausibility and filtering the results by scores or *p*-values. Modern genome annotation pipelines use a variety of gene prediction programs and combine their results using heuristic methods. The resulting number of predicted genes is usually considerably larger than the number of genes for which there is expression evidence. For example, the Ensembl gene annotation (release 64, http://www.ensembl.org/) for the GRCh37 version of the human reference genome includes 46 993 predicted human genes. In contrast, only 26 976 genes have supporting experimental evidence (Figure 1, Supplementary Figure S1).

Clearly, many gene predictions are false positives. Assessing the rate of false-positive prediction is usually difficult because of the absence of appropriate negative controls; using imprecisely matched negative controls quickly leads to wrong estimates of statistical significance. Furthermore, the definition of a ‘false positive’ is not trivial and will depend on the specific genomic analysis being performed. When assessing the ability to identify *Alu* repeats and other evolved interspersed elements, it is reasonable to consider any such identification in a negative control as a false positive. In the case of gene prediction, the ideal negative control would have all the properties and characteristics of the genome, except it should be guaranteed to lack genes and other functional elements. Even then, any random sequence could potentially be processed and interpreted by the transcriptional and translational machinery of the cell; short ORFs arising at random could potentially yield biologically active peptides. While even such ‘random’ predictions may be of interest, it is still important to assess how frequently they arise for each gene prediction algorithm and for each type of negative control used. A common practice in other areas is the use of ‘decoys’. For example, in proteomics the use of randomly permuted peptides in MS/MS spectra database searches can assist in the computation of a robust e-value (26). There is currently no standard method or protocol to define a set of sequences to be used as negative controls for genome analysis. A common practice is to use intergenic regions as negative control, under the assumption that they lack functional elements. A widely used method used to generate decoys in sequence analysis is to ‘shuffle’—or permute—the sequence being studied. This method involves random base permutation, typically performed in such a way to preserve the observed dinucleotide composition. Shuffling may be performed globally or locally. The advantage of global shuffling is its simplicity, but it may lose many important properties of the DNA sequence, e.g. G+C content inhomogeneities. Other methods have been used to generate sequences representing the ‘background’. For example, in an analysis of short ORFs in *Drosophila* (27), the authors used as negative controls a pool of ‘reverse’ small ORFs defined as sequences from a stop codon to the next start codon, and following a similar size distribution.

We present here a novel method for generating ‘decoy’ artificial DNA sequences with the same characteristics of intergenic sequences in terms of composition, complexity and presence of interspersed elements. We started by performing a detailed compositional analysis of the reference genome; this yielded a multifaceted set of models describing the characteristics of intergenic sequences.

Drawing parameters from these rich models, our sequence generation method has two stages. In the first stage, a ‘base’ sequence is produced with compositional characteristics similar to the unique portion of real, intergenic sequences. In the second stage, this base sequence is ‘bombarded’ with artificially evolved interspersed repeats and low complexity sequences. The resulting artificially generated sequences are nearly indistinguishable from real intergenic regions. Finally, we used the artificial sequences to test the specificity of programs for predicting coding and non-coding genes, and for identifying interspersed repeats.

Our method can generate an unlimited amount of artificial sequences mimicking real genomic sequences for any species available in the UCSC Genome Database, and it can be trained for other species given adequate genome information.

## MATERIALS AND METHODS

### Genome data used

We obtained genomic sequence and annotations, from the UCSC Genome Database (http://genome.ucsc.edu), for the following species—human: hg19 (3); chimpanzee: panTro3 (9); cat: felCat4 (12); chicken: galGal3 (8); cow: bosTau4 (14); dog: canFam2 (10); elephant: loxAfr3 (http://www.broadinstitute.org/scientific-community/science/projects/mammals-models/elephant/elephant-genome-project); fugu: fr2 (28); guinea pig: cavPor3 (http://www.broadinstitute.org/scientific-community/science/projects/mammals-models/guinea-pig/guinea-pig-genome-project); horse: equCab2 (13); lizard: anoCar2 (29); marmoset: carJac3 (http://genome.wustl.edu/genomes/view/callithrix_jacchus); medaka: oryLat2 (30); mouse: mm9 (7); opossum: monDom5 (31); orangutan: ponAbe2 (32); panda: ailMel1 (33); pig: susScr2 (11); platypus: ornAna1 (34); rabbit: oryCun2 (http://www.broadinstitute.org/scientific-community/science/projects/mammals-models/rabbit/rabbit-genome-project); rat: rn4 (35); rhesus: rheMac2 (36); sheep: oviAri1 (http://www.sheephapmap.org/); stickleback: gasAcu1 (http://www.broadinstitute.org/models/stickleback); tetraodon: tetNig2 (http://www.genoscope.cns.fr/externe/tetranew/); *Xenopus tropicalis*: xenTro2 (37); zebra finch: taeGut1 (38); zebrafish: danRen7 (The Danio rerio Sequencing Project http://www.sanger.ac.uk/Projects/D_rerio/); *Ciona intestinalis*: ci2 (39); purple sea urchin: strPur2 (40); *Anopheles gambiae*: anoGam1 (41); bee: apiMel2 (42); *Drosophila melanogaster*: dm3 (6); *Caenorhabditis elegans*: ce3 (43).

### Interspecies comparison

To define intergenic and intronic regions in hg19, we combined the gene annotations in the tracks knownGene.txt, ensGene.txt and vegaGene.txt from the UCSC Genome Database. Intergenic ranges represent all genomic regions excluding combined gene ranges. Intronic ranges are the intersection of all regions inside gene coordinates after excluding any overlap with annotated exons. For each region, we used the LiftOver tool (44) to search the respective region in the other genomes for which LiftOverChain is available. The total spans of these regions are reported in Supplementary Table S1.

We obtained the respective sequence for all regions for each species and computed the tetramer composition per species and stratified into five GC content bins [0%–37%, 37%–39%, 39%–42%, 42%–45% and 45%–100%] that have approximately equal total genomic span. We combined all tables to compute the principal component analysis (PCA) showed in Figure 3.

**Figure 2.**
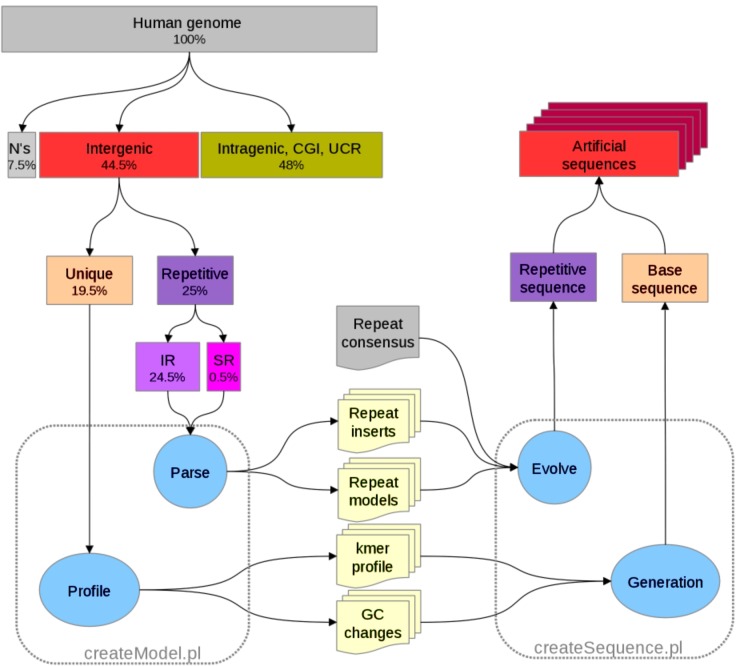
Overview of the algorithms. Our development consists of two stages: (1) training (left side): the fraction of the genome remaining after masking of functional and repetitive regions is analyzed for *k*-mer and GC content. Repeats are also modeled, separated into interspersed (IR) and simple sequence repeats (SR); (2) generation (right side): a base sequence is generated using the *k*-mer and GC profiles. Artificially evolved repeats are then inserted into the base sequence to create a new artificial sequence.

**Figure 3.**
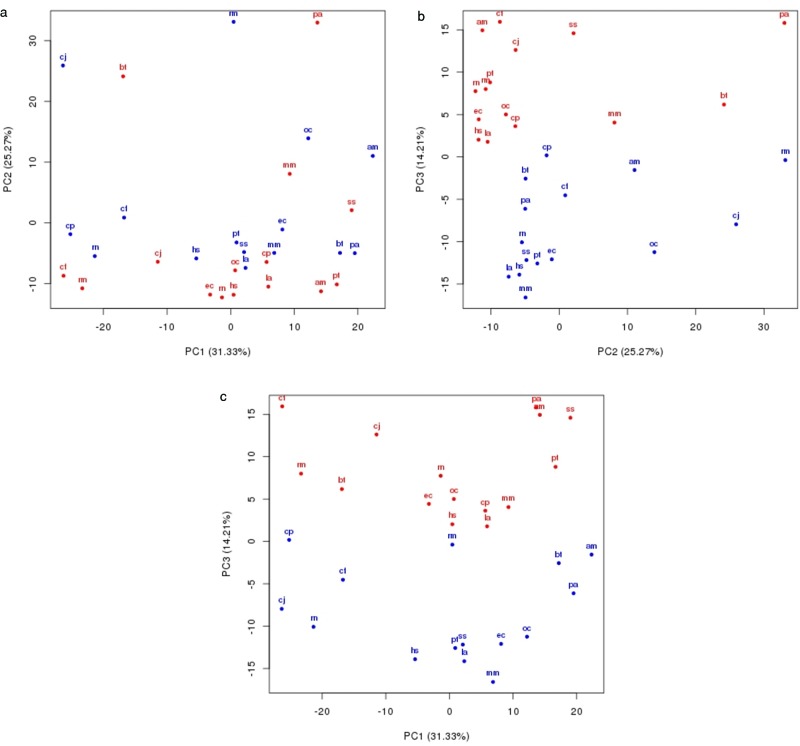
Multi-species PCA. We compared the GC-binned tetramer composition of orthologous sequences in human (hs), chimpanzee (pt), cow (bt), dog (cf), elephant (la), guinea pig (cp), marmoset (cj), horse (ec), mouse (mm), orangutan (pa), panda (am), pig (ss), rabbit (os), rat (rn) and rhesus (rm) for intergenic (blue) and intronic (red) regions.

### Intergenic region profile

We created profiles summarizing GC content characteristics of the human genome, excluding the non-chromosomal sequences (chrUn, mitochondria, haplotype-specific regions and diverse unassigned regions). The first step is to compute the local GC content homogenized in 10 kb bins: we compute the local GC content in non-overlapping 1 kb windows and average them in 10 kb sections. The GC content is classified into five bins as described above.

We removed from the reference genome sequence (3.1 Gb) all fragments annotated as known genes, non-coding RNA and pseudogenes (UCSC tracks: knownGene.txt, ensGene.txt, vegaGene.txt, vegaPseudoGene.txt), CpG islands (UCSC track: cpgIslandExt.txt), interspersed repeats (from RepeatMasker annotation, UCSC file chromOut.tar.gz), simple repeats (from TRF annotation, UCSC file chromTrf.tar.gz) and ultraconserved regions (UCSC tracks: phastConsElements46way.txt filtered by region >100 bp and multiz46way.txt filtered for regions >100 bp and score >0.65). After removing all these, 574 Mb remained (17% of the genome). For each sequence, we computed a table containing the *k*-mers in 1 kb long non-overlapping windows and classified each window by GC content. This table contains the probabilities of each *k*-mer to be followed by each possible nucleotide [A, C, G, T]. These probabilities are derived empirically from the frequency of each such event, observed in the reference genome sequence. We also compared the GC content of consecutive non-overlapping windows and used these to compute the transition probabilities between different GC levels. We obtained annotations of repetitive elements from the RepeatMasker output from the UCSC Genome Database. For each interspersed repeat in intergenic regions, we recorded the type, family, percentage of divergence from the corresponding consensus sequence, insertions and deletions, size and number of fragments. We also assigned each repeat to a GC bin according to its position in the genome. We further recorded the frequency of repeats that inserted into other repeats.

We extracted annotations of simple sequence repeats from TRF output. For each repeat, we recorded the consensus monomer, size, divergence, insertions and deletions, and assigned it to the respective GC bin according to its position in the genome.

We provide the models for all species used in this study in the project website.

### Sequence generation

The method to generate a new artificial sequence has two main steps: (i) generate a base sequence of the size required and (ii) addition of simple and interspersed repeats randomly selected from observed genome distributions. Each repetitive sequence is artificially evolved from the corresponding repeat family consensus. Repeats are then inserted into the base sequence in random location and orientation based in the local GC content as observed in the training dataset. To avoid inserting older repeats into younger ones, the most diverged repeats are inserted first, using the divergence from the consensus as an approximation of repeat age.

### Generation of repetitive elements

For each selected repeat, we use the sequence derived from the consensus reported in RepBase rel. 20090120 (45). First the consensus is trimmed to an equivalent length as seen in the annotation, then a series of mutation stages are used to artificially evolve it. The first stage deletes random positions in the sequence; the second stage mutates random positions as transition and transversion events; in the third stage insertions are added at random positions. Each time a position is selected, the probability of inserting a repeat is evaluated considering the frequency of the adjacent *k*-mers produced. One additional step evaluates the fragmentation probability: if the element is fragmented, then a new younger element is selected from the repeat insertion table.

### Generation of low complexity repeats

Our algorithm expands the monomer consensus to the desired length, then applies a series of mutation stages (deletions, transitions, transversions and insertions) in a similar way to the artificial evolution of repetitive elements described before.

### Sequence validation

Real genomic sequences have certain constraints on composition and complexity. Our training method relies on composition properties of the sequences; complexity properties are not explicitly modeled. After generating artificial sequences, we computed various complexity metrics and compared these with the expected values for real intergenic sequences. Since our artificial sequences are (by definition) unique due to the random generation process, we could not compare them with real sequences using traditional alignment methods. Therefore, we evaluated the sequences using alignment-free methods, in terms of base composition, dimer skews, complexity and repeat content. We performed three types of comparisons between real sequences, artificial sequences and shuffled sequences:

1. We visualized individual real, artificial and shuffled sequences using the GESTALT Workbench (46).
2. We compared (i) 80 sequences of 100 kb each randomly selected from the hg19 genome after discarding functional regions (genes, pseudogenes, CpG islands, ultraconserved elements), (ii) the same intergenic sequences permuted independently using the dinucleotide composition conservation with the Altschul–Erickson shuffle algorithm (47), and (iii) a set of simulated sequences of equal size, G+C content and repetitive fraction, generated using tetramer models and window size of 1000 bases. We built a matrix with the calculated values for sequence composition and complexity (see below) and analyzed it using both PCA and cluster analysis in the statistical program R.
3. We compared 10 randomly selected real intergenic sequences to 100 artificial sequences modeled after each real sequence (with similar G+C content and repetitive fraction using tetramer models and window size of 1000 bases) and to 100 dimer permutations of each real sequence. We then compared the distributions of sequence composition and complexity (see below) and repeat content (fraction of sequence in SINEs, LINEs, LTR elements, DNA transposons and simple repeats, as tallied by RepeatMasker).

#### Sequence composition

We used three composition-based measures (Equations (1–3)): G+C content (*gc*), G/C skew (*gcs*) and CpG ratio (*cpg*). G+C content and CpG ratio are common descriptors of the sequence composition, showing significant variation along the chromosomes in a genome:

(1) \begin{equation*} {gc = \frac{{n_G + n_C }}{{n_A + n_T + n_C + n_G }}} \end{equation*}

(2) \begin{equation*} {gcs = \frac{{n_G - n_C }}{{n_G + n_C }}} \end{equation*}

(3) \begin{equation*} {cpg = \frac{{n_{CG} }}{{n_G \cdot n_C }}}, \end{equation*}

where *n_X_* is the total count of base *X*.

#### Sequence complexity

We calculated the complexity of the sequences using five different methods (Equations (4–8)). The Wootton–Federhen complexity *cwf* (48) and the entropy of the symbols *ce* (49) measured the complexity as function of the monomer composition. For the entropy of symbols in a Markov model *cm* (49) and the linguistic complexity *cl* (50) we used word lengths of 6, 8 and 12, calculating the complexity as a function of words observed in relation to all the possible words in an alphabet. Additionally, we quantified the compressibility of the sequence *cz* with the Zlib library (http://www.zlib.net/). Zlib uses two algorithms, the first step is a LZ77 compression which maps and masks repeated patterns, and the second step is symbol encoding with Huffman trees. The level of compression is inversely related to the complexity of the sequence: low complexity sequences, like simple repeats, have higher compression factor than most coding sequences:

(4) \begin{equation*} cwf = \frac{1}{N} \cdot \log _4 \frac{{N!}}{{\prod\limits_{i \in ACGT} {n_i !} }}, \end{equation*}

where *N* is the length and *n_X_* is the total count of base *X*.

(5) \begin{equation*} ce = - \sum\limits_{i \in ACGT} {\left( {\frac{{n_i }}{N} \cdot \log _4 \frac{{n_i }}{N}} \right)}, \end{equation*}

where *N* is the length and *n_X_* is the total count of base *X*.

(6) \begin{equation*} cm = - \sum\limits_{i = 1}^{4^m } {\left( {\frac{{m_i }}{{N - m - 1}} \cdot \log _{4^m } \frac{{m_i }}{{N - m - 1}}} \right)}, \end{equation*}

where *N* is the length and *m* is the word length and *m_t_* is the total count of the *t* word.

(7) \begin{equation*} cl = \frac{{\sum\limits_{i = 1}^m {V_i } }}{{\sum\limits_{i = 1}^m {V_{\max (i)} } }}, \end{equation*}

where *V_t_* is the total distinct words with *i* length and *V_max_* is the maximal number of observed words of length *i*.

(8) \begin{equation*} cz = \frac{{Z(s)}}{{S(s)}}, \end{equation*}

where *S*(*s*) is the size in bytes of the sequence *s* and *Z*(*s*) is the size in bytes after compression.

#### Cluster analysis

We performed a cluster analysis to validate the similarity of artificial sequences to real and shuffled intergenic regions using the composition and complexity measures described above. The data matrix was analyzed with the Partitioning Around Medoids (PAM) method from the package *cluster* in R (51). The PAM method is an improved version of *k*-means clustering algorithm with additional diagnostic information related to the cluster solution. This diagnostic method is known as the silhouette score with a value span from 0 to 1, with a score of 0 given to elements that are probably classified in the wrong cluster and 1 as the element is most likely to be in the correct cluster. Since we are comparing three sequence classes, we used an expected group value of 3.

### Repeat detection

As described before, we performed three analyses for repeat detection. The first analysis was intended to estimate the real false-positive rate for PClouds (52) and RepeatMasker in artificial sequences generated without any repetitive elements. We used a set of 100 intergenic regions of 100 kb each from the human genome; each sequence was permuted independently (conserving dinucleotide composition) using the Altschul–Erickson shuffle algorithm (47). We generated a similar set of artificial sequences without repeats using the same G+C content as the real intergenic region and generated with *k*-mer size of 8 and window size of 1000.

We ran RepeatMasker open-3.2.7 using the default parameters and ‘-species human’ flag. To test PClouds, we trained the model as recommended by the authors, then we ran PClouds v0.9 with the following configuration as used by the authors in the original report with C10 parameters: [OligoSize → 16, COPYTHRESHOLD → 2, ENDTHRESHOLD → 10, STEP1THRESHOLD → 20, STEP2THRESHOLD → 200, STEP3THRESHOLD → 2000, CALCHUNCKSIZE → 10000000, GENOMESIZE → 3200000000, WindowSize → 10, PercentCutoff → 80]. We used custom Perl scripts to parse the outputs and report the total bases detected as repetitive.

The second and third test challenged specific models for detection of *Alu* and MIR repeats. We generated a set of sequences with a G+C content between 40% and 60% (representing the average G+C in the genome) and inserted artificially generated *Alu* repeats in a gradient of sequence fraction from 10% to 90%. We generated 10 sequences of each category and computed an average. We ran RepeatMasker with default parameters and ‘-species human’ flag and PClouds trained with the 1000 *Alu* sequences used by the authors in the original report. Those sequences were obtained randomly from the RepeatMasker output for hg17. After training, we tested PClouds with word size of 8, 10, 12, 14 and 16 using C10 parameters. We used custom Perl scripts to parse the outputs and report the total bases detected as TP, TN, FP and FN. MIR tests were performed in a similar way.

### Gene prediction

#### Coding genes

We used Genscan v1.0 (16), Twinscan v3.5 (17) and Augustus v2.0.3 (18) as *ab initio* gene prediction programs, using default parameters and the trained models for detection of human genes, as provided by the authors with their respective programs. We ran each program against the set of sequences with repeat regions masked.

#### Non-coding genes

We used Infernal v1.2 (21) to predict putative non-coding RNAs using default parameters. We selected all models from Rfam v9.1 (53) that were built including human sequences in the model alignment.

### Transcriptional footprints

We used FEAST (25) with default parameters to identify regions with transcriptional footprints—a combination of orientation skews indicative of transcriptional activity.

### Data access

The models and software can be accessed from the project website www.repeatmasker.org/garlic/. We implemented the algorithm in the Perl language for multiple platform compatibility. All the code is released as Open Source under the GPLv3.0 license.

## RESULTS

### A statistical model of the non-functional genome

We model the genome as being composed of three classes of sequence: (i) sequences under functional or mutational constraints (typified by transcribed regions), (ii) sequences that arose by duplication but are largely unconstrained (typified by intergenic interspersed repeats), and (iii) a background or ‘base’ sequence. The functionality of introns is under debate (54,55) but they are at the very least mutationally biased (24). Since our goal is to characterize and recreate unconstrained sequences, we conservatively excluded class (i) in its entirety, including all introns. We studied the compositional characteristics of classes (ii) and (iii), and the mutational spectrum of class (ii) sequences (repeats) relative to their respective consensus sequences.

Based on the available annotation of the human genome, we identified 574 Mb of ‘base’ sequence (17% of the genome) left after removing all fragments annotated as genes, pseudogenes, CpG islands, ultraconserved sequences and repetitive sequences (see the Materials and Methods section). Of the 3.1 Gb in the human reference genome (GRCh37, hg19), 7.5% is ambiguous sequence (gaps and heterochromatin) and 48% is annotated as transcribed (including introns). The remaining 44.5% of the genome is composed of intergenic sequence, of which 25% is repetitive and 19.5% is unique. We define this last fraction (intergenic and unique) as the ‘base’ sequence.

We analyzed the base sequence and computed the frequencies of all observed *k-*mers in fixed-size non-overlapping windows stratified by G+C content. Since the choice of these parameters may affect the quality of the sequence generated, we tested different values of *k* (4,6,8,10,12) and window length [200, 500, 1000, 2000, 5000] but we did not observe significant differences between the sequences produced (data not shown). For our tests, we chose models with *k =* 4 and window size of 1000 as default. We also computed the G+C content transition frequency between adjacent windows. This is a measure of how frequently a region shifts from one G+C class to another. These changes are common in the genome, typically triggered by the presence of repetitive elements or other functional constrains such as CpG islands. A general overview of the processes described above is presented in Figure 2.

### Intergenic and intronic regions are compositionally distinct

In the genome, there are two classes of largely non-functional regions that could be used for model training: intergenic and intronic regions. Introns are known to be mutationally biased (24); their composition is therefore expected to differ from that of unconstrained intergenic sequence. We verified the existence of such a difference by comparing the intergenic and intronic regions in the human genome—representing 1.2 Gb and 403 Mb, respectively—and their orthologous regions in 14 other species (Supplementary Table S1). We computed the tetramer frequencies in those regions, binning by G+C content, and compared the intergenic and intronic profiles of the 15 genomes simultaneously by PCA. The third principal component clearly distinguishes between intergenic and intronic regions (blue and red, respectively, in Figure 3).

### Generation of artificial sequences

**Figure 4.**
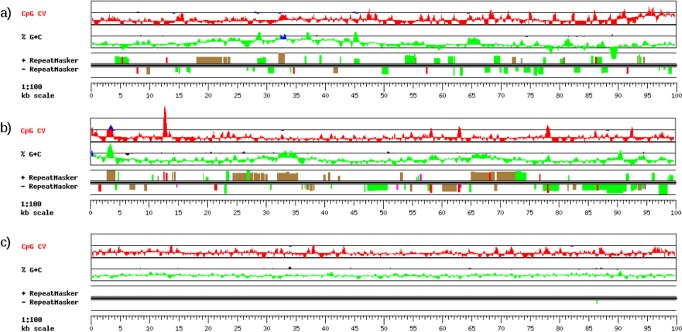
GESTALT comparison of artificial, real and shuffled sequences. (**a**) Artificial sequence, (**b**) Intergenic region chr4:104640972–104740972, (**c**) Intergenic region chr4:104640972–104740972 after dimer permutation. The elements in RepeatMasker are: LINEs in green, SINEs in red/pink, LTR, DNA transposable elements and others in brown.

**Figure 5.**
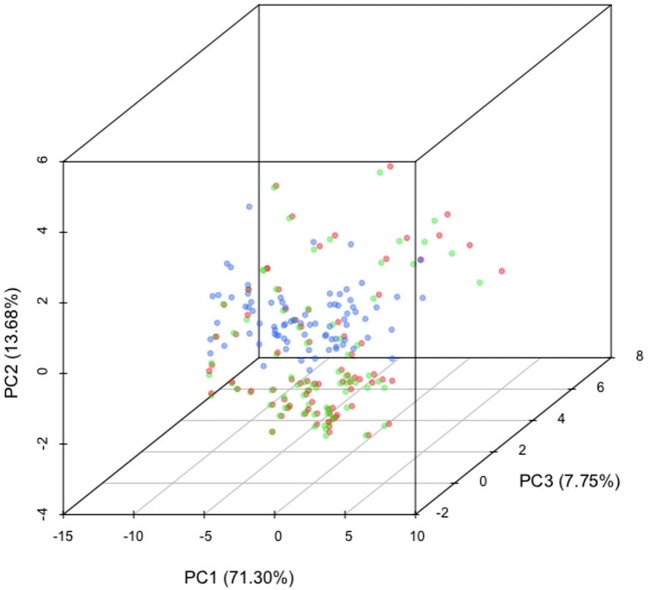
Principal component analysis of composition and complexity measures. We compare 100 sequences with 100 kb each for artificial sequences (blue), selected intergenic regions (green) and their respective dimer permutation of intergenic sequences (red).

We implemented a workflow for generating artificial sequences or arbitrary length and with similar characteristics to those of unconstrained intergenic regions of the genome. In its simplest form, our workflow requires specifying just the target species and the desired length of the artificial sequence. Additional optional parameters include *k*-mer size, window size, and the desired repetitive fraction of the sequence. If unspecified, the latter is modeled from the real genome distributions. Visual comparison of the generated artificial sequences to real intergenic regions with similar G+C content and repetitive fraction shows that our algorithm captures the complexity of the G+C content across the sequence and the diversity of interspersed repeats (Figure 4). In contrast, local shuffling scrambles the sequence in interspersed repeats, leading to unrealistic results. We further applied alignment-free comparison methods to verify that the composition and complexity of the repeat-masked artificial sequences is comparable to real intergenic sequences (for details see the Materials and Methods section). We created for each sequence a numerical vector including all its quantified properties and visualized the vectors in a multiscale projection after dimension reduction with PCA (Figure 5). The dispersal range of artificial sequences is equivalent to that of real intergenic sequences—if somewhat less diverse—showing high compositional similarity. We also used cluster analysis methods on the numerical matrix of the composition and complexity values to test whether the sequences generated can be distinguished from the real sequences. After application of the PAM method with three expected clusters, the clusters showed mixed elements of artificial, real and shuffled sequences with an average silhouette value of 0.77, a value considered as stable (Supplementary Figure S2).

The sequences generated have similar composition and complexity to that of real intergenic sequences, but clearly show a larger diversity in complexity. Repetitive content is similar in proportions as expected in the real intergenic sequences, while shuffled sequences are essentially devoid of repetitive content—typically including just a small fraction of simple repeats and none of the other repeat classes (Supplementary Figure S3).

### Evaluation of repeat detection methods

We compared the performance of PClouds (52) and RepeatMasker when analyzing (i) the expected false-positive rate for repetitive regions modeling in the human genome, (ii) *Alu*-specific model accuracy and (iii) *MIR*-specific model accuracy. For the first test we selected 100 intergenic sequences of 100 kb each from the human reference and dimer permuted in 1000 non-overlapping windows. We generated a similar number of 100 kb sequences using those intergenic regions as model for expected G+C level in 10 kb windows and generated using *k-*mer size of 8 in non-overlapping 1000 bp window size. We trained the PClouds model using the protocol described in the original article with word size of 16 and C10 parameters over the 23 human chromosomal sequences, and then used PClouds to identify repetitive region in our sequence test set. We analyzed all the test sequences using RepeatMasker with default parameters. Since we included no repeat elements while generating the sequence, any prediction represents a false positive. We computed the false-positive rate per base (FPR) as

**Figure 6.**
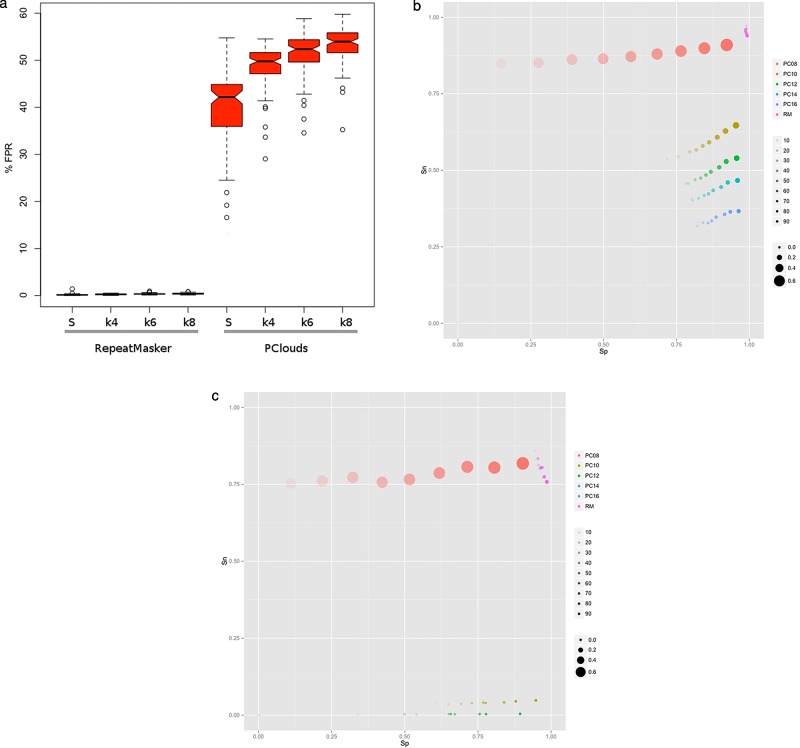
Repeat identification benchmarks. (**a**) False positives expected in dimer-permuted sequences and synthetic sequences without repeats, (**b**) *Alu* tests, (**c**) MIR tests. For (b) and (c), each point represents the average sensitivity (Sn) and specificity (Sp). Colors represent the program (PC = PClouds, RM = RepeatMasker) and word size used for PClouds (8, 10, 12, 14, 16). The transparency of the color denotes the amount of repetitive sequence in each set (10%, 20%, … 90%) and the size of the point the average FPR. Each sequence set includes 10 artificial sequences of 100 kb each generated with *k*-mer size of 8 and window size of 1000 with a G+C content of 40–60%.

\begin{equation*} {\rm FPR} = {\rm FP}/{\rm L}, \end{equation*}

where FP is the total bases identified as repetitive and L the total length of the sequence. Figure 6a summarizes the results obtained. In dimer-permuted sequences, RepeatMasker's FPR is very low (median of 0.13%), while PClouds’ FPR is significant (median of 42.16%). For artificial sequences we observed higher median values for different *k*-mer sizes in PClouds (49.77%, 52.37% and 53.96% for *k*-mer of 4, 6 and 8, respectively), while the median FPR for RepeatMasker is consistently below 0.39%.

For the *Alu* specific benchmarks we generated a series of 100 kb artificial sequences; we used a G+C content between 40% and 60% and created 10 independent sequences with {10, 20, 30, … 90%} repetitive content exclusively of artificially generated *Alu* sequences. We trained PClouds with the same 1000 *Alu* sequences reported in the original article with word size of {8, 10, 12, 14, 16} and C10 parameters. We ran RepeatMasker with default parameters and post-processed the results selecting only predicted *Alu* regions. In the artificial sequences, we know the exact positions where *Alu* were inserted, therefore we can compute Specificity (Sp), Sensitivity (Sn) and false-positive rate (FPR) values as

\begin{equation*} {\rm Sp} = {\rm TN}/({\rm TN} + {\rm FP}) \end{equation*}

\begin{equation*} {\rm Sn} = {\rm TP}/({\rm TP} + {\rm FN}) \end{equation*}

\begin{equation*} {\rm FPR} = {\rm FP}/({\rm TN} + {\rm FP}), \end{equation*}

where TP are True Positives, FP are False Positives, TN are True Negatives and FN are False Negative bases.

Figure 6b shows our results for RepeatMasker and PClouds. PClouds' results are affected by the repetitive fraction and word size selected. PClouds’ authors (52) reported that two thirds of the human genome is repetitive. We found that PClouds has a false-positive rate of up to 60%, higher than the 12.6% reported by the authors, and that it depends on the *k*-mer used and the sequence composition even for models specific for *Alu*s or MIRs. In contrast, RepeatMasker's FPR is lower and stable (<0.4%) and with high sensitivity (>90%) and specificity (>75%)

Similarly to the *Alu* benchmark, we created a set of test sequences for MIR and analyzed them using the same strategy. We present in Figure 6c our results and we observed a similar performance of RepeatMasker compared to PClouds, RepeatMasker has good specificity (>92.10%), sensitivity (>71.29%) and low FPR (<7.9%). PClouds was affected again by the repetitive content and word size.

### Evaluation of gene prediction methods

**Figure 7.**
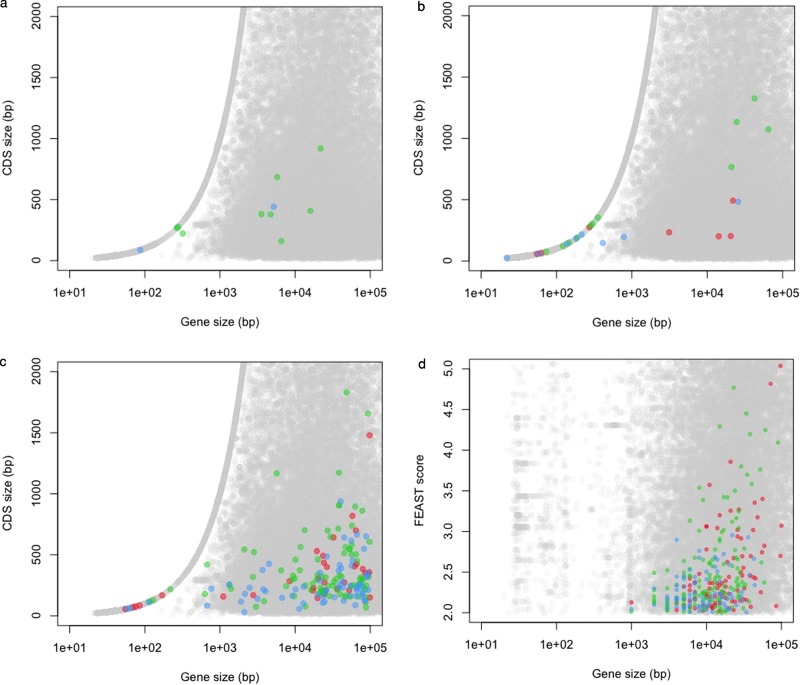
Coding and transcribed size distribution of predicted genes. Predictions in artificial (blue), intergenic (green) and dimer permuted intergenic sequences (red) are compared with known genes (gray) for (**a**) Augustus, (**b**) Genscan, (**c**) Twinscan and (**d**) FEAST.

We challenged three gene prediction programs: Genscan (16), Twinscan (17) and Augustus (18) with artificially generated sequences lacking, by design, any real gene structures. Since some transposable elements include protein-coding regions, it is customary to mask repeats prior to gene prediction. We therefore repeat-masked our artificial sequences. The resulting base sequence, interrupted by gaps where the repeats used to be, is known not to include any coding regions that have been subjected to evolutionary selection. Nevertheless, all the programs tested reported complete genes, including transcription signals, exons and introns, and coding regions (Figure 7). The longest gene prediction in artificial sequences spanned 90 kb, with a predicted protein product of ∼300 aa. We used NCBI blast (56) against the NR database of non-redundant peptides (accessed April 2012) to test whether the newly predicted coding regions have any similarity to known protein sequences. As expected, this comparison found no significant hits (e-value < 0.1). When studying artificial sequences, Augustus predicted fewer unselected gene structures than Genscan and Twinscan (0.2, 6.5 and 0.8 genes/Mb, respectively). The three programs predicted fewer gene structures in shuffled sequences (no predictions, 3.3 and 0.7 genes/Mb, respectively).

We similarly tested how frequently Infernal (21) predicts non-coding RNAs in the artificial sequences. We used the 198 models available for human-related sequences in Rfam (53). Infernal did not predict any non-coding RNAs in the analyzed intergenic, artificial and shuffled sequences with a significance e-value < 0.01.

Finally, we challenged FEAST (25) to find regions with transcription potential (Figure 7d). The FEAST scores observed in the artificial sequences follow a similar distribution as the scores from real intergenic sequences. FEAST predicted 9.3 transcribed regions/Mb in artificial sequences and none in shuffled sequences. This false-positive rate may be inflated since FEAST scores represent compositional and repeat content skews between the opposite DNA strands, and artificial sequences show some excess compositional skews relative to real sequences.

## DISCUSSION

Richard Feynman wrote on his blackboard in 1988, ‘What I cannot create, I do not understand’: the artificial reproduction of an object reflects our knowledge about it. We thus set out to create a new algorithm that can produce sequences indistinguishable from the real non-functional intergenic regions from a genome. With the initial draft of the human genome, it was estimated that approximately 5% of the human genome is likely to be functional (3). This figure has been revised upward based on significant new experimental evidence (54). The current annotation of the human genome includes 56 563 genes with experimental evidence annotated in Gencode v16 (57). Although there are almost twice as many hypothetical, predicted or putative genes reported, many of them are expected to be false-positive predictions. The experimental validation of all these predicted genes may prove to be difficult since genes (or specific transcripts thereof) may be expressed at too low levels, for too short windows of time, or in too specific a context (e.g. very limited stages of development or in response to very specific stimuli). Conversely, many of the *ab initio* gene predictions may be false positives. It is difficult to estimate a real false-positive rate without a realistic negative control.

Based on composition analysis and the distortions introduced by the absence of repeats we demonstrated the inadequacy of simple permutation-based methods for generating negative control sequences: the dimer shuffle methods do not produce sequences that are realistic enough to serve as negative control for gene prediction. In addition, both local and global shuffling remove the presence of repeats, making it impossible to correctly test repeat detection programs. We also showed that intronic and intergenic sequences have very different compositions (Figure 3). One explanation for this difference is that the regions have a different evolutionary pressure; introns have a strong bias in composition (24) and repeat orientation (25). Therefore, intronic regions are not adequate as negative control. In the case of intergenic regions, we identify two main problems with using annotated intergenic regions for training and testing gene identification tools:

1. Presumed intergenic regions may include yet-unidentified genes and functional elements. Despite being frequently classified as dispensable DNA, intergenic regions include fragments that are highly conserved between divergent species, but of unclear function (58). Additionally, a series of reports over the past few years have indicated that a much larger portion of mammalian genomes is transcribed (59,60) and even has been claimed that up to 80% of the human genome is biochemically functional (54). Uncharacterized functional elements may deviate in composition or other relevant sequence features from the random distributions required for negative controls.
2. The amount of sequence available for each species is limited, reducing the ability to perform multiple testing. Just one quarter of the 3 billion bases in the human genome are considered intergenic and non-functional (3).

Previous efforts in DNA sequence simulation have mainly focused on analysis of coding regions. Programs such as Seq-Gen (61), Gsimulator (62) and many others start from a gene sequence and perform an artificial evolution process, adding mutations and modeling the conservation of the coding portion. All these programs are specific for coding portions in small genome scales, and are not implemented to work with large genomes like human. There are many selective pressures on coding sequences that constrain the way in which they can mutate, and the mutation rate is variable between regions. Therefore these methods are not directly applicable to modeling the evolution of intergenic sequences to produce the desired negative controls. More elaborate efforts evolve a sequence using predefined mutation rates (63), then permute the sequences to obtain a new sequence with similar composition (64). In this case, typical elements as repeats are missing and we can expect the same bias as observed in our shuffled sequences. Similar to the first stage of our algorithm, previous work generated synthetic ‘pseudo-genome’ sequences by training a 15-state hidden Markov model (65). Our artificial sequences reflect the expected frequency distributions of longer word sizes and include artificially evolved repeats. The resulting sequences are visually similar to real intergenic sequences. In contrast, the permuted sequences clearly are devoid of repetitive elements, even when they conserve composition and complexity properties. In our training process, we explicitly capture the composition properties of intergenic sequences as the *k*-mer frequencies observed in each of the five GC content bins. We do not model sequence complexity explicitly. Nevertheless, the model produced by our training steps can produce complex sequences similar to those observed in the genome, with minor differences in G+C skew, compressibility and complexity quantified in Markov models. These minor discrepancies arise by the random process of selection and insertion of repeats.

Our artificial sequences mimic in composition and complexity the large, non-functional portion of the genome. We tested the sequences produced by the models comparing real intergenic, artificial and shuffled sequences in terms of composition and complexity as described above. Using these metrics as dimensions in PCA and PAM/Silhouette clustering, we observed a good similarity between classes. We also tested the diversity of composition and complexity features in sequences produced by repeated simulations and shuffling of 10 real intergenic sequences. All sequences produced were similar in composition and complexity (gc, cpg, cwf, ce, ct, cl). In contrast with shuffled sequences, the artificially generated sequences also contained a diverse family of elements like interspersed repeats (LINEs, SINEs, LTR elements and DNA transposons), and low complexity sequences based on real models. Our program also has an option to report how exactly each repeat sequence was constructed, indicating where each mutation occurred. Our method does not yet model certain types of repeats for which there is some discordance between the identifier of the consensus in Repbase and the annotation by RepeatMasker, and some specific types like satellite and telomeric sequences. These are region-specific sequences, infrequently found interspersed elsewhere in the genome, and which arise via a generative process different from that of transposons and retrotransposons.

Even randomly generated sequences may include by chance sequences that, if introduced to a living cell, might perform a biological function. The probability of such events will depend on the size and complexity of the required signal for functional activity: short motifs like a transcription factor binding site will frequently arise by chance, while complete gene structures encoding meaningfully functional proteins will rarely do so. Thus, for gene prediction we expect most observations in random sequences to be false positives. Our method for computationally generating artificial genomic sequences enhances the ability to estimate the true frequency with which such positive events happen.

Applications for complete genome simulations are not limited to assessing gene and repeat discovery methods. For example, genome-size simulations can be used to test mapping efficiency of short reads (66). Other researchers (67) criticized the results of the ENCODE project and proposed a ‘Random Genome Project’ as the missing negative control. Our algorithm can be used to produce such genome-size sequences. In addition to the applications described above, our method for generating artificial genome sequences can be useful for assessing a variety of other computational biology algorithms, including prediction of *cis*-regulatory motifs, estimation of composition fluctuations in chromosomes, or genome fractal properties.

## SUPPLEMENTARY DATA

Supplementary Data are available at NAR Online.

Supplementary Data
