# Supplementary material for: Realistic artificial DNA sequences as negative controls for computational genomics
Source: Nucleic Acids Res. 2014 May 6;42(12):e99. doi: 10.1093/nar/gku356 (PMC4081056; doi:10.1093/nar/gku356)
Supplement: Supplementary Data [file supp_42_12_e99__index.html]

Realistic artificial DNA sequences as negative controls for computational genomics — Supplementary Data 

# Realistic artificial DNA sequences as negative controls for computational genomics

## Supplementary Data

**Files in this Data Supplement:**

- Supplementary Data
