# Supplementary material for: Realistic artificial DNA sequences as negative controls for computational genomics
Source: Nucleic Acids Res. 2014 May 6;42(12):e99. doi: 10.1093/nar/gku356 (PMC4081056; doi:10.1093/nar/gku356)
Supplement: Supplementary Data [file supp_gku356_nar-02747-met-k-2013-File016.pdf]

Realistic Artificial DNA Sequences as Negative Controls for Computational Genomics

Supplementary Data.

Authors:

Juan Caballero, Arian F. A. Smit, Leroy Hood and Gustavo Glusman*

Affiliation:

Institute for Systems Biology, 401 Terry Ave. N, Seattle, WA 98109, USA

Emails:

jcaballero@systemsbiology.org

asmit@systemsbiology.org

lhood@systemsbiology.org

Gustavo@systemsbiology.org

* To whom correspondence should be addressed.

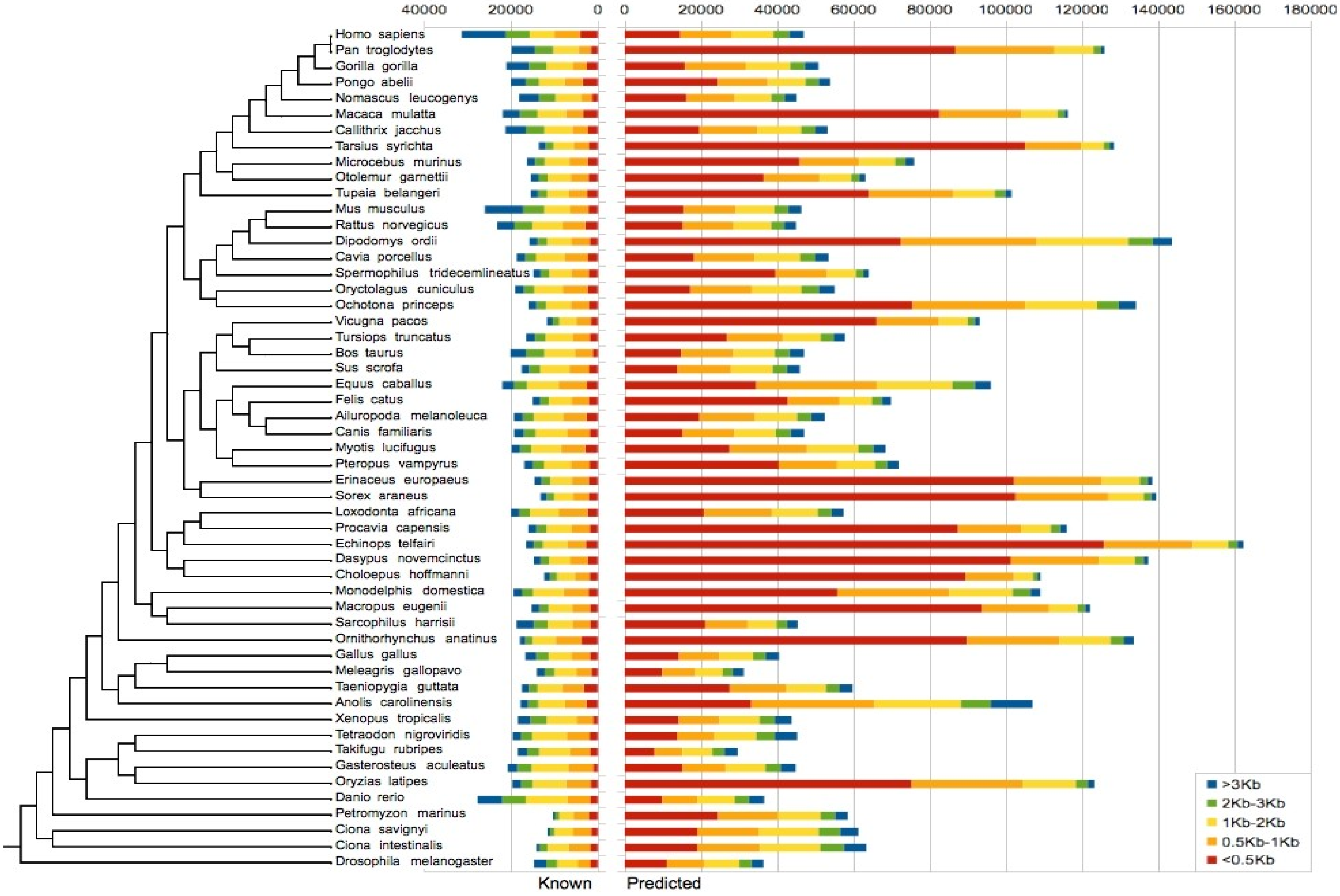

Supplemental Figure 1. Total number of known and predicted coding genes in Ensembl 64 for human and other species. The gene size is determined by the total length in bases of each CDS.

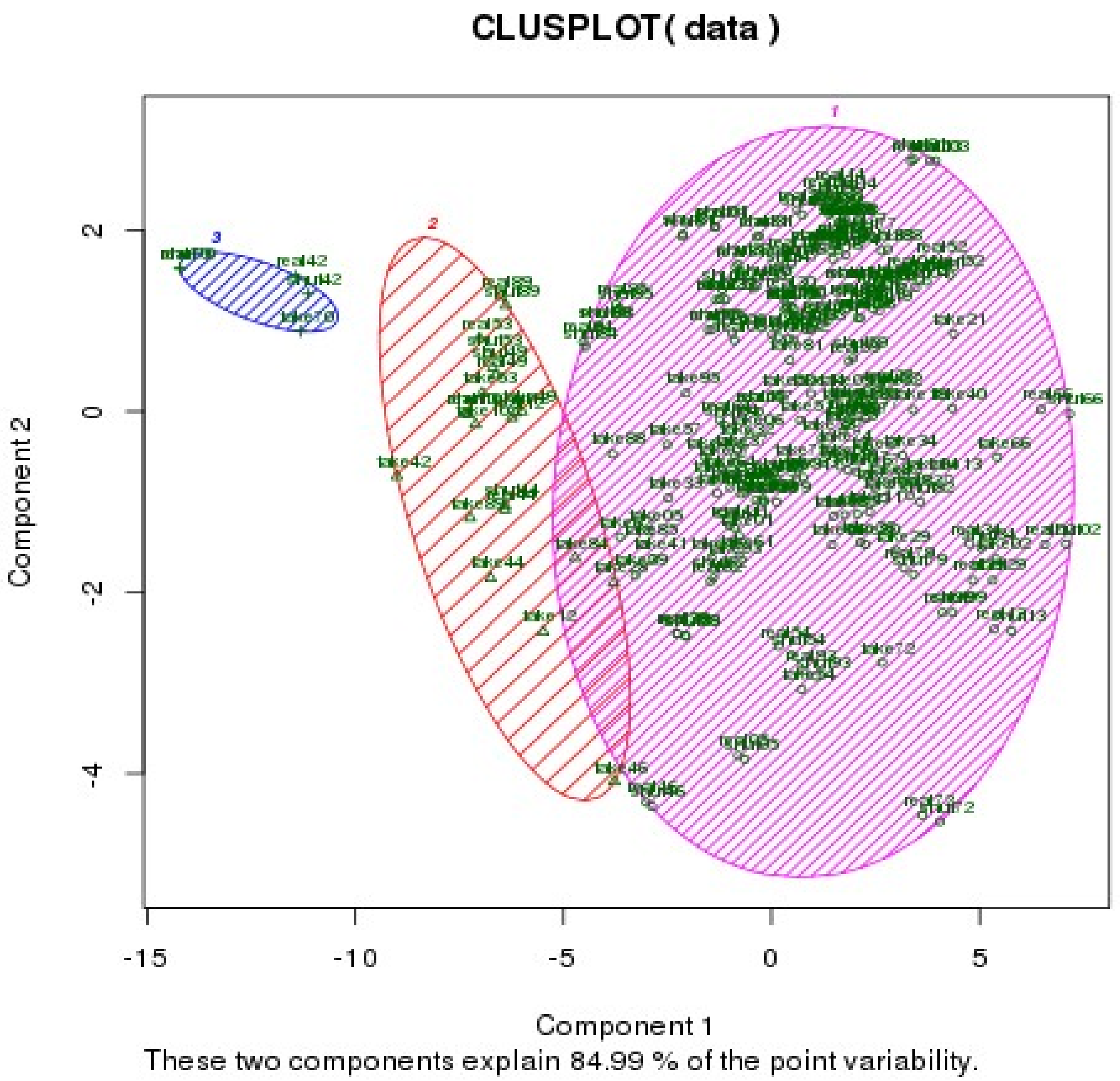

Supplemental Figure 2a. Cluster analysis of sequences. Custering analysis was performed in 100kb - 100 human intergenic regions, a dimer permutation of those sequences and 100 artificial sequences (kmer=8, window=1000) labeled as real, shuffle and fake respectively. Each sequence was evaluated in terms of composition and complexity, as described in the main text. Numerical values were used as independent dimensions for the Partitioning Around Medoids (PAM) method in R. Since we are comparing three sequence classes, we used an expected group value of 3, showed as the color ellipses.

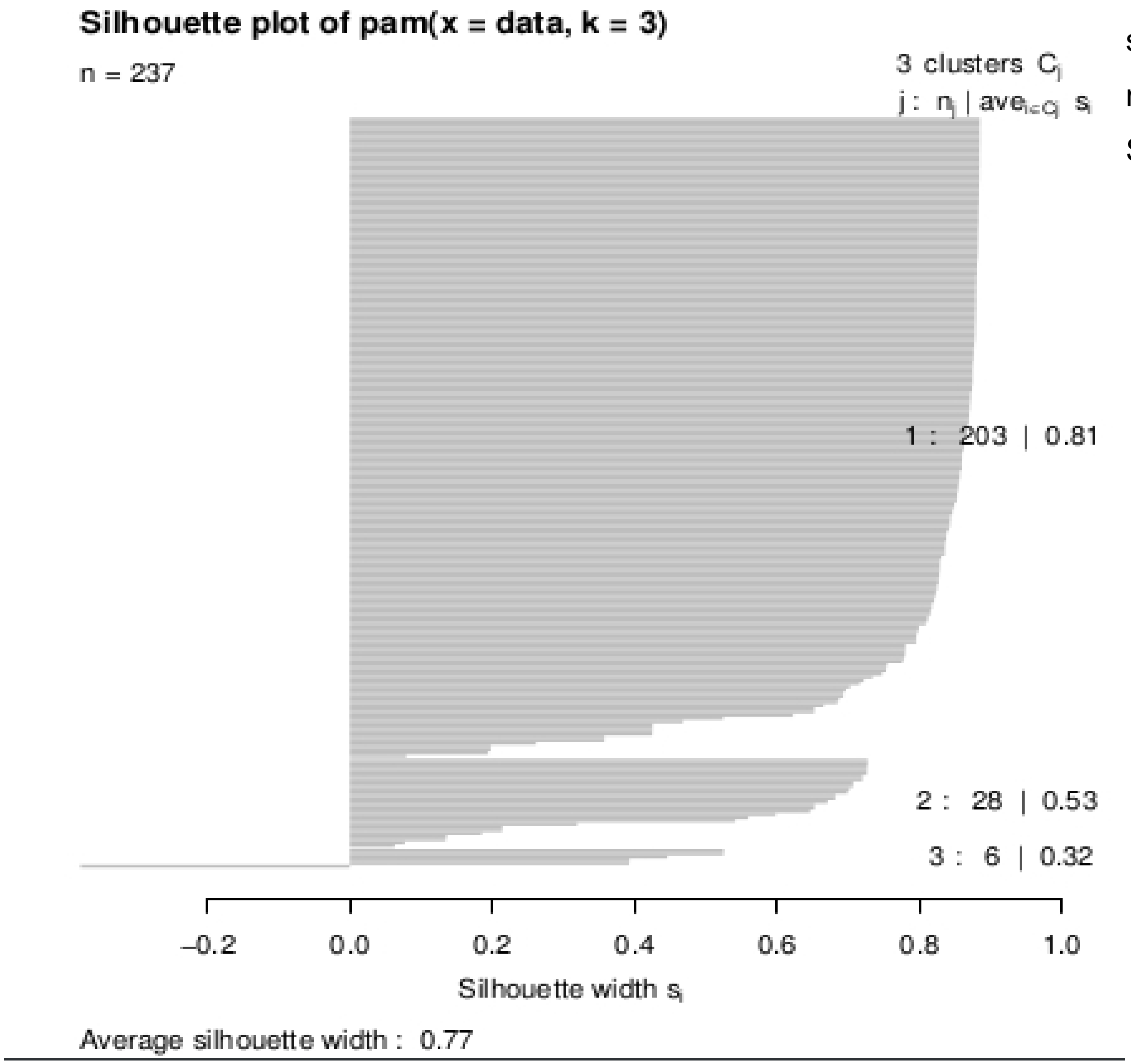

Supplemental Figure 2b. Cluster analysis of sequences. Classified sequences by the PAM method were evaluated for Average Silhouette Score of each group.

Supplemental Figure 3a. Composition, complexity and repetitive percentage distributions for 100 artificial sequences (A) created after human intergenic region in chr4:176097250-176197250 with similar length, G+C content and fraction of repetitive elements and 100 dimer permutations of the same sequence (S). The red line shows the value in the original intergenic region.

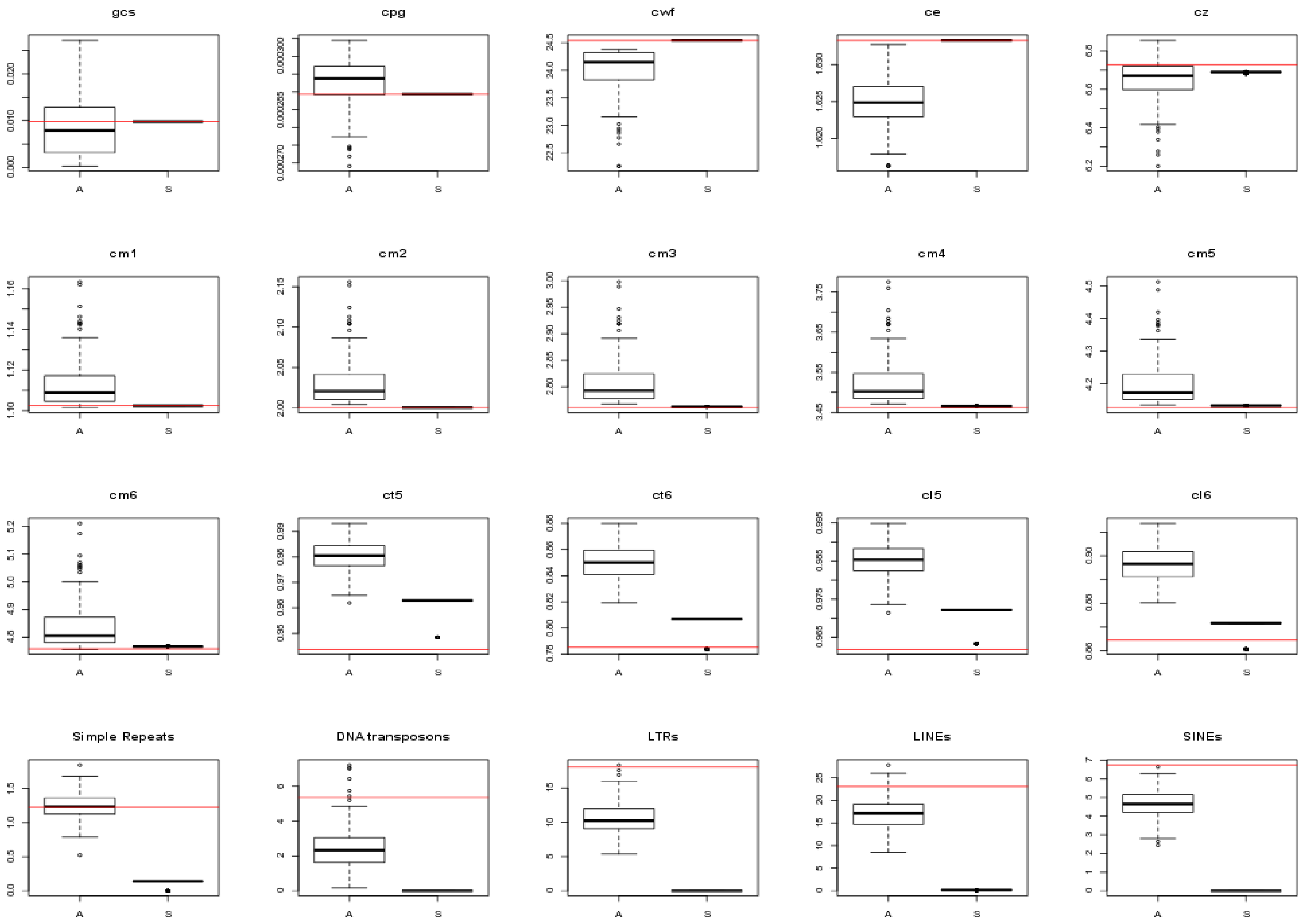

Supplemental Figure 3b. Composition, complexity and repetitive percentage distributions for 100 artificial sequences (A) created after human intergenic region in chr12:61662934-61762934 with similar length, G+C content and fraction of repetitive elements and 100 dimer permutations of the same sequence (S). The red line shows the value in the original intergenic region.

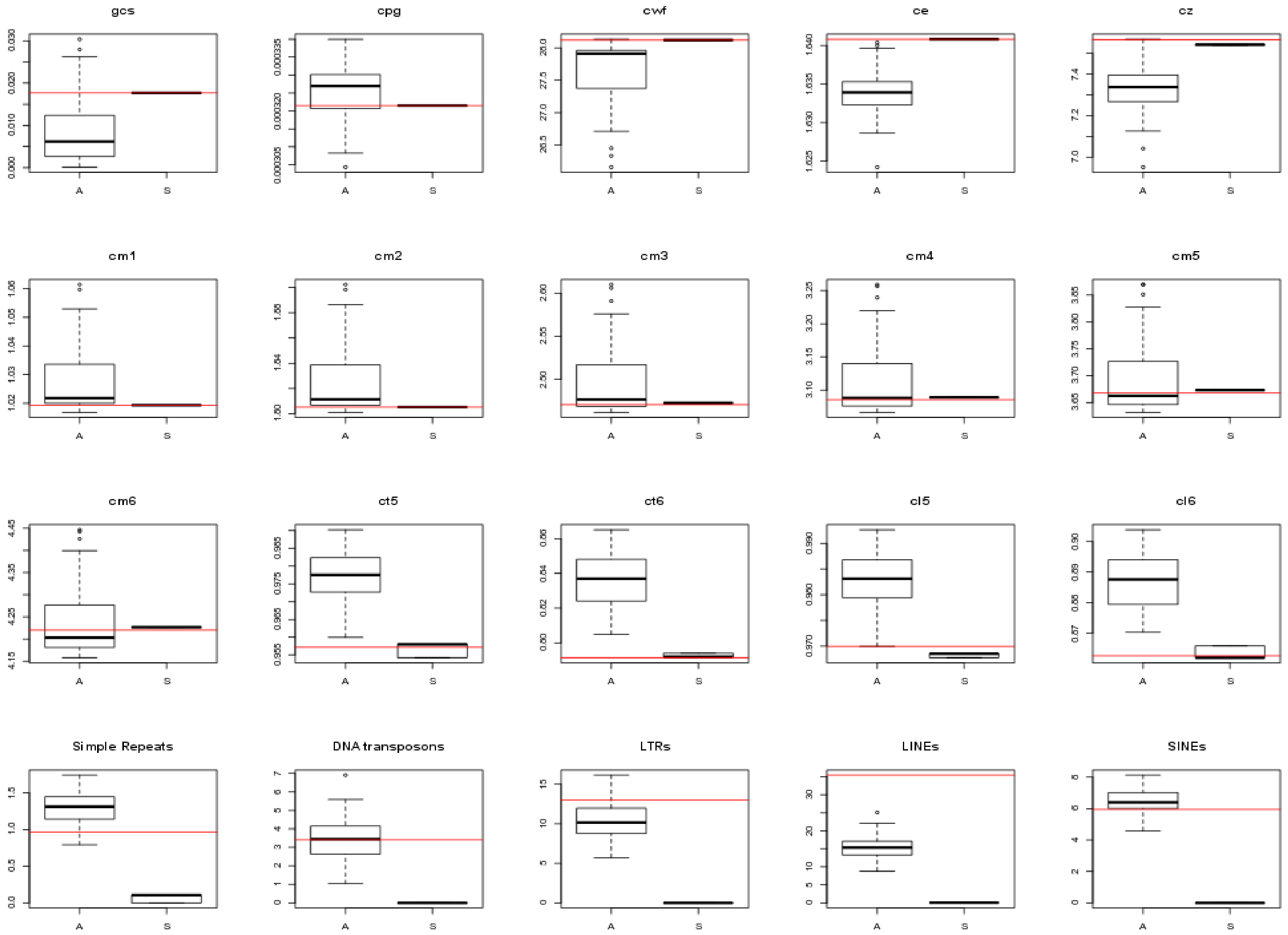

Supplemental Figure 3c.Composition, complexity and repetitive fraction distributions for 100 artificial sequences (A) created after human intergenic region in chr14:42539946-42639946 with similar length, G+C content and fraction of repetitive elements and 100 dimer permutations of the same sequence (S). The red line shows the value in the original intergenic region.

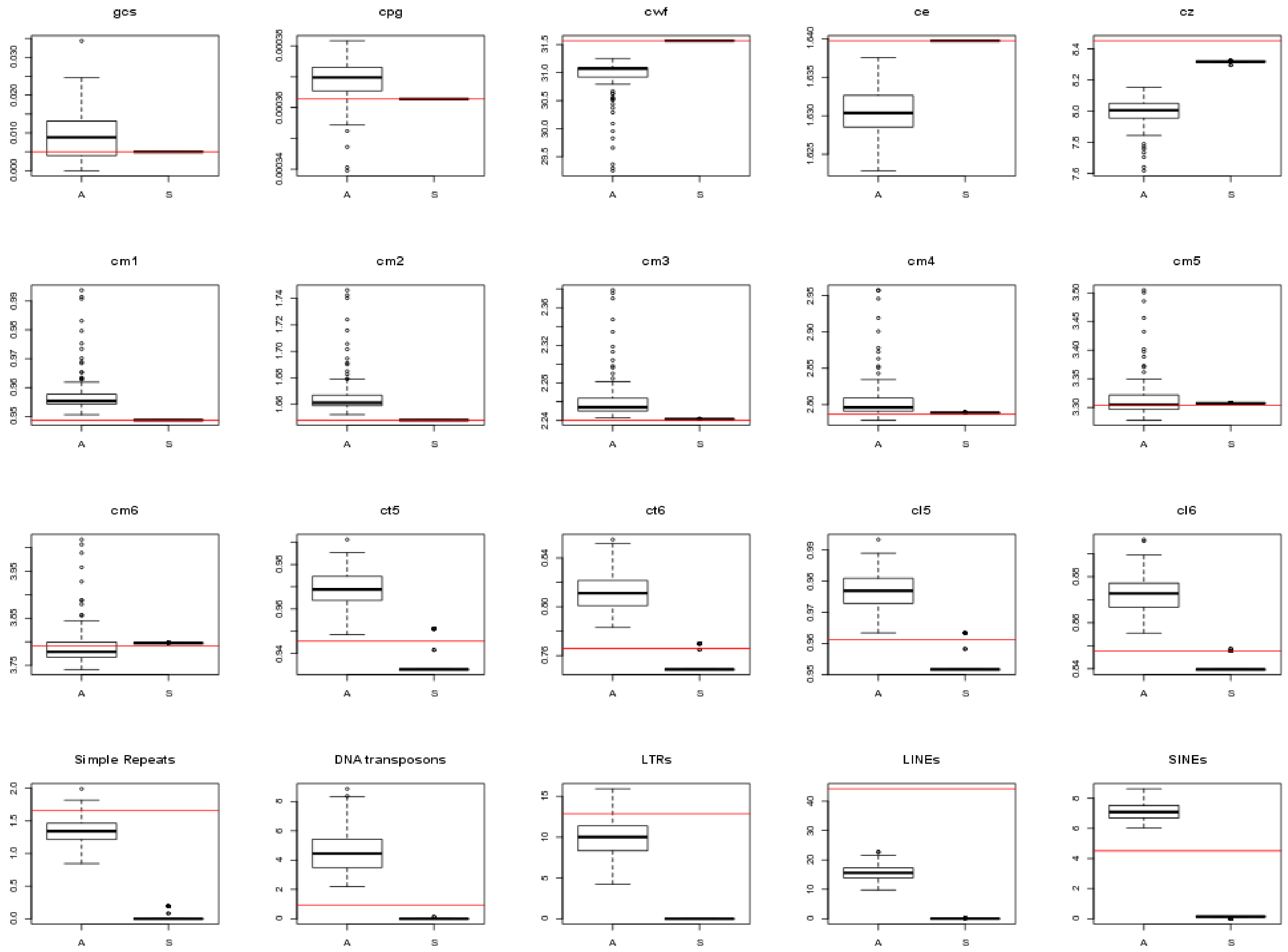

Supplemental Figure 3d. Composition, complexity and repetitive percentage distributions for 100 artificial sequences (A) created after human intergenic region in chr2:57297819-57397819 with similar length, G+C content and fraction of repetitive elements and 100 dimer permutations of the same sequence (S). The red line shows the value in the original intergenic region.

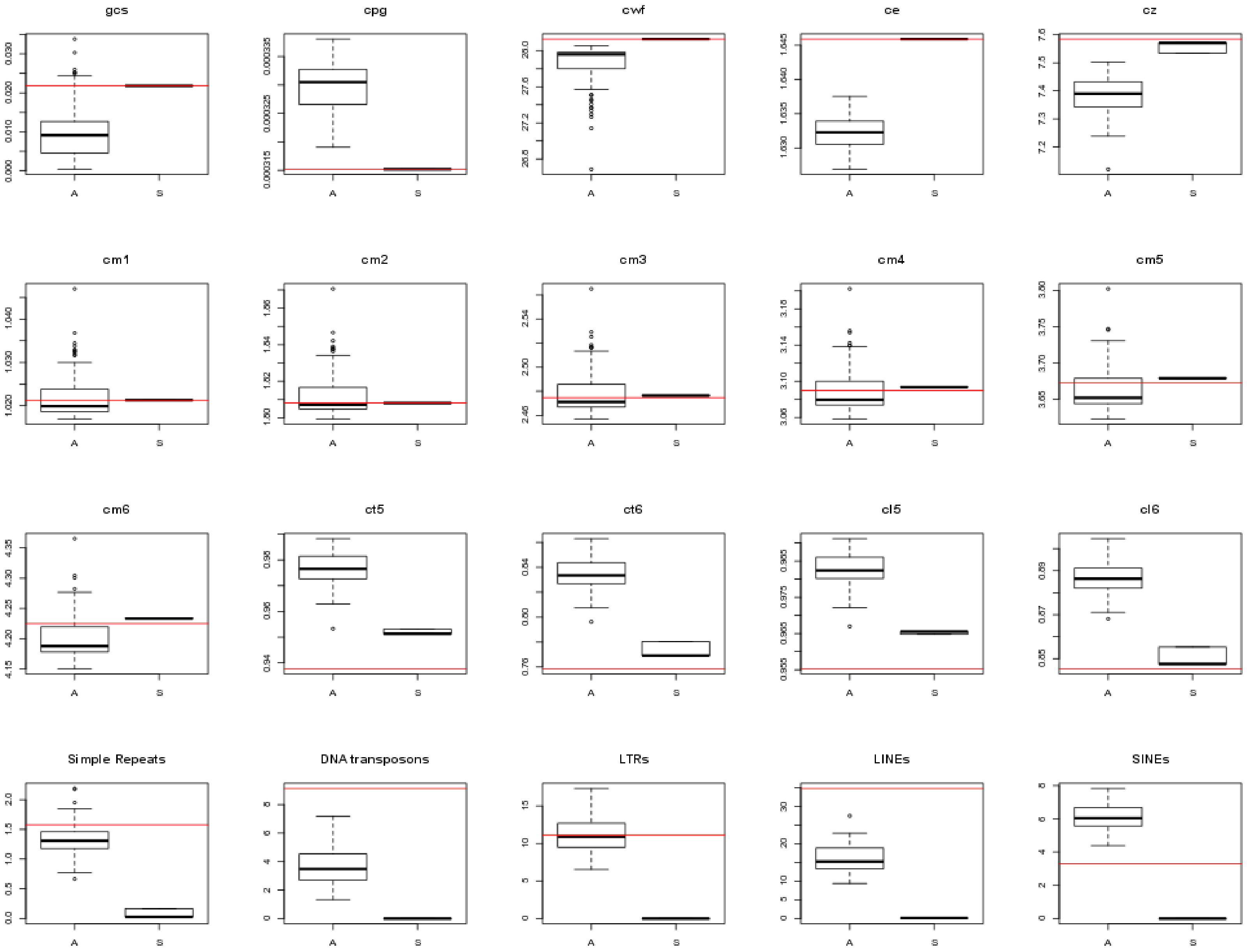

Supplemental Figure 3e. Composition, complexity and repetitive percentage distributions for 100 artificial sequences (A) created after human intergenic region in chr3:164576982-164676982 with similar length, G+C content and fraction of repetitive elements and 100 dimer permutations of the same sequence (S). The red line shows the value in the original intergenic region.

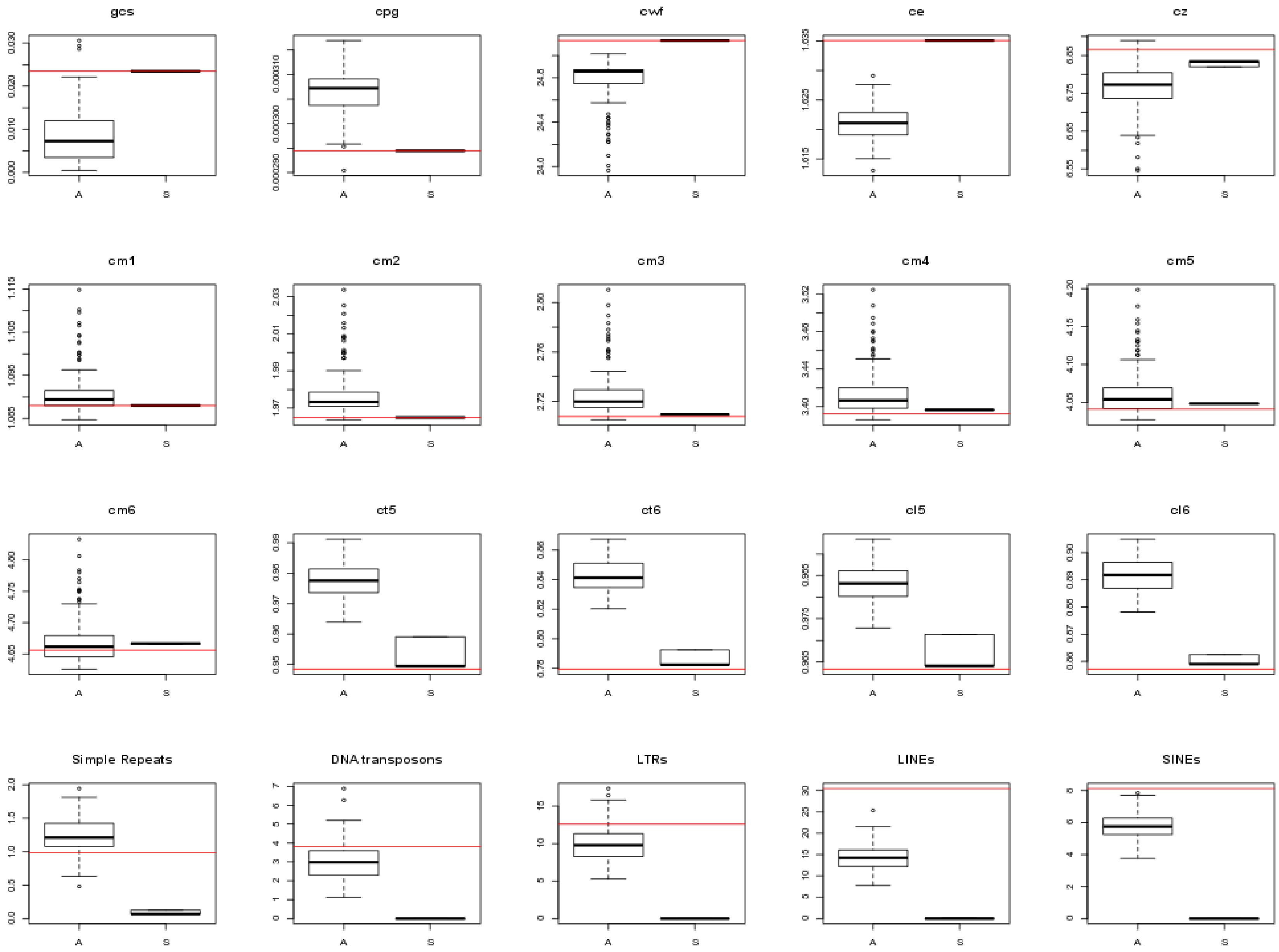

Supplemental Figure 3f. Composition, complexity and repetitive percentage distributions for 100 artificial sequences (A) created after human intergenic region in chr11:24104709-24204709 with similar length, G+C content and fraction of repetitive elements and 100 dimer permutations of the same sequence (S). The red line shows the value in the original intergenic region.

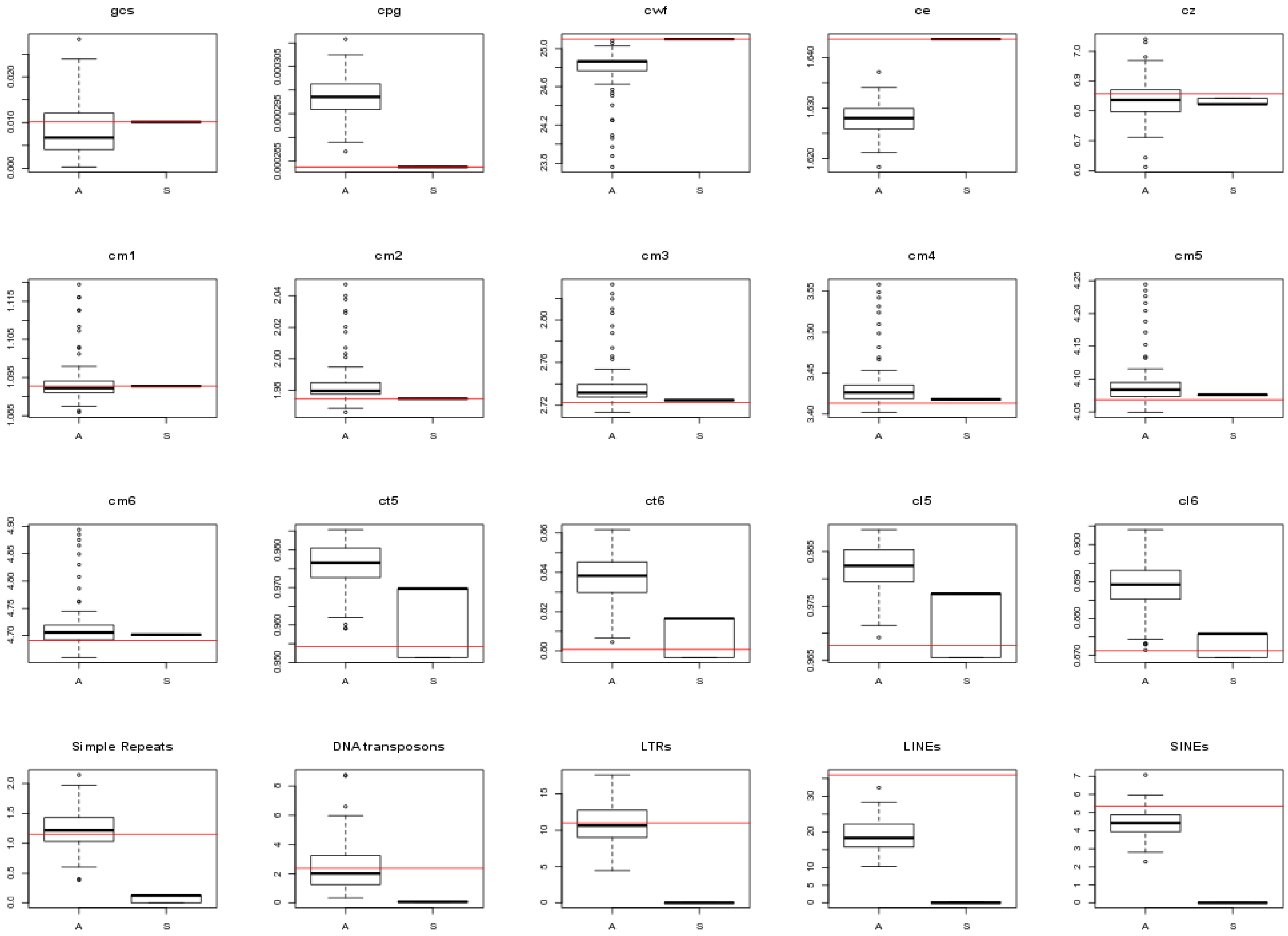

Supplemental Figure 3g. Composition, complexity and repetitive percentage distributions for 100 artificial sequences (A) created after human intergenic region in chr14:48576793-48676793 with similar length, G+C content and fraction of repetitive elements and 100 dimer permutations of the same sequence (S). The red line shows the value in the original intergenic region.

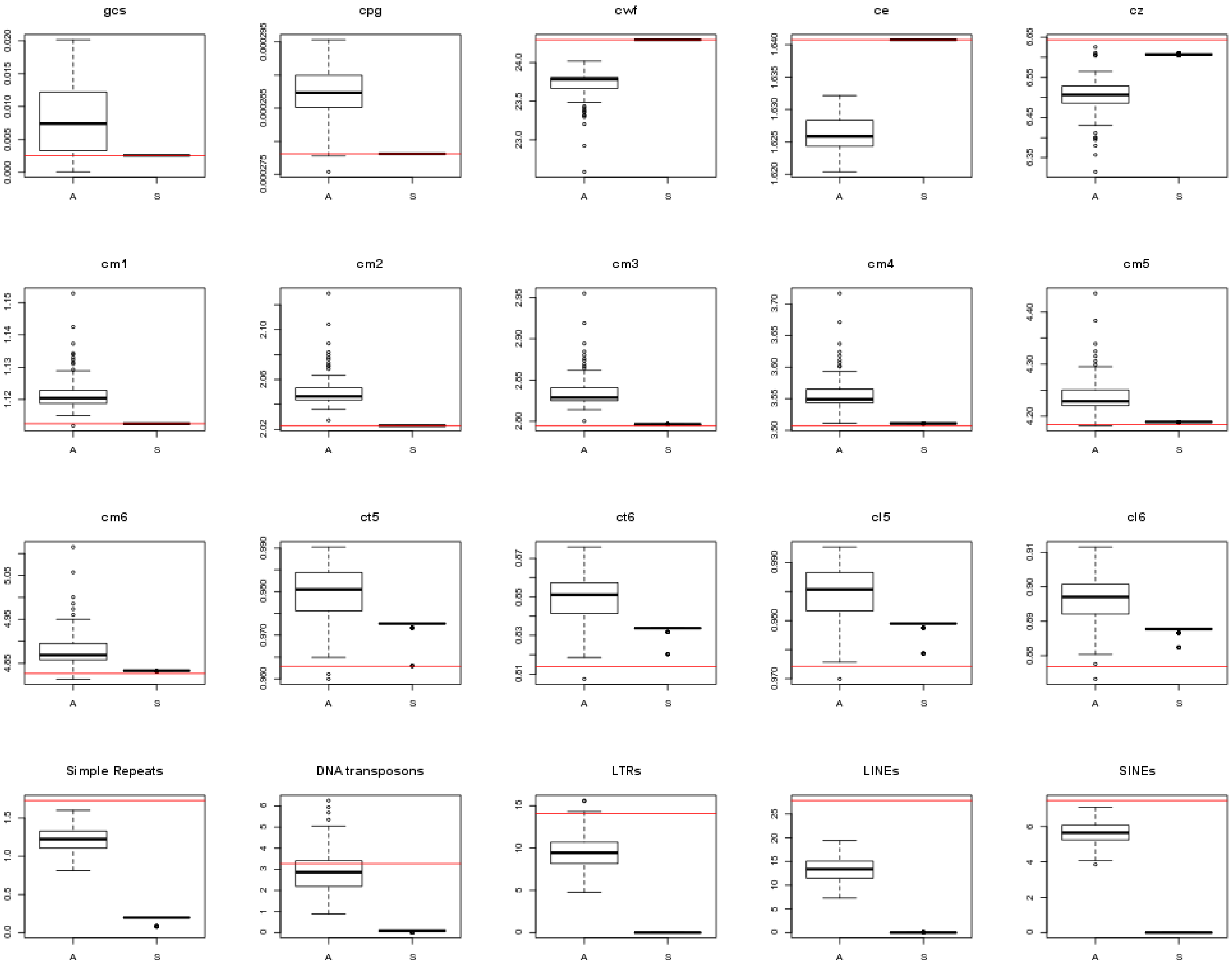

Supplemental Figure 3h. Composition, complexity and repetitive percentage distributions for 100 artificial sequences (A) created after human intergenic region in chr2:82849841-82949841 with similar length, G+C content and fraction of repetitive elements and 100 dimer permutations of the same sequence (S). The red line shows the value in the original intergenic region.

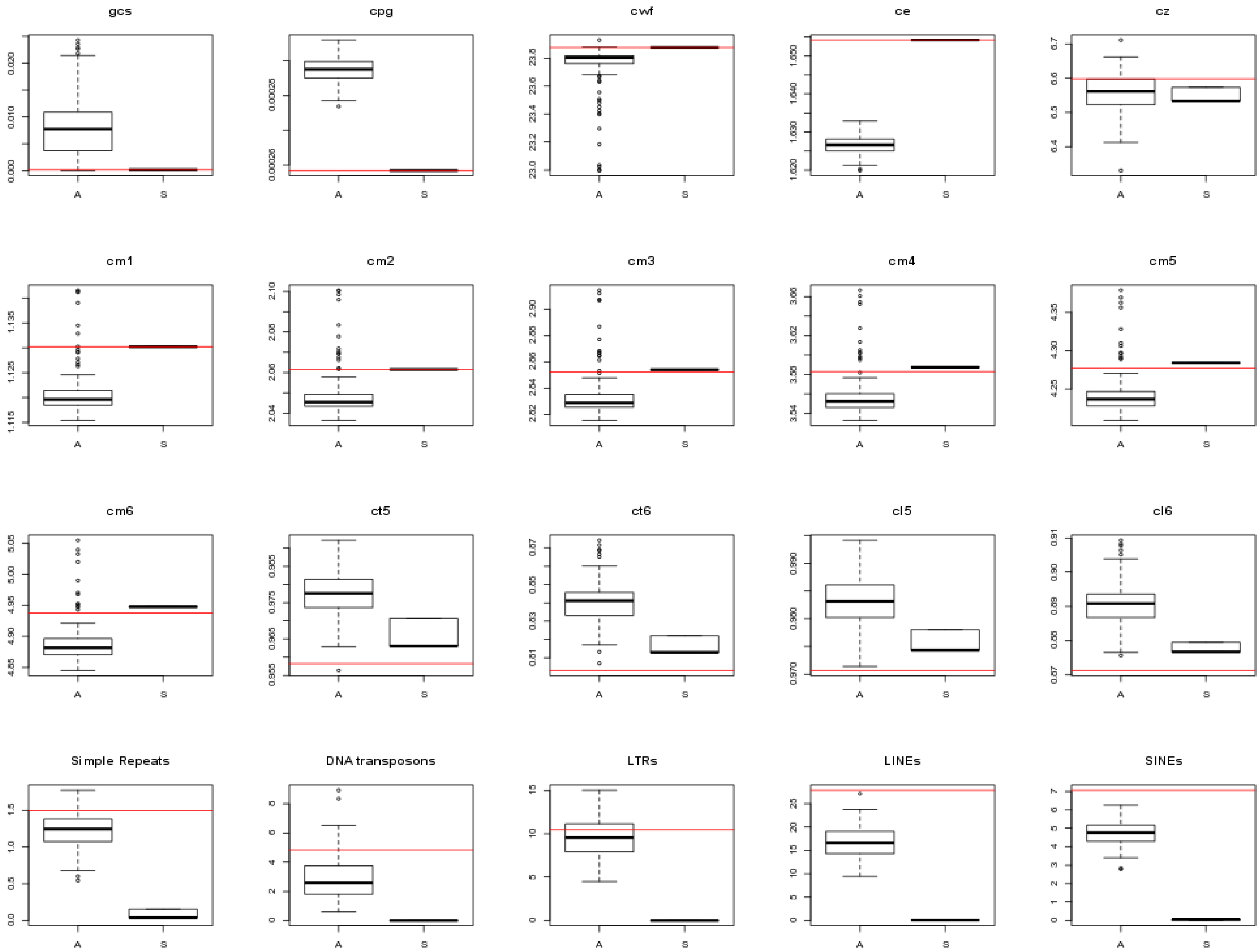

Supplemental Figure 3i. Composition, complexity and repetitive percentage distributions for 100 artificial sequences (A) created after human intergenic region in chr1:238789803-238889803 with similar length, G+C content and fraction of repetitive elements and 100 dimer permutations of the same sequence (S). The red line shows the value in the original intergenic region.

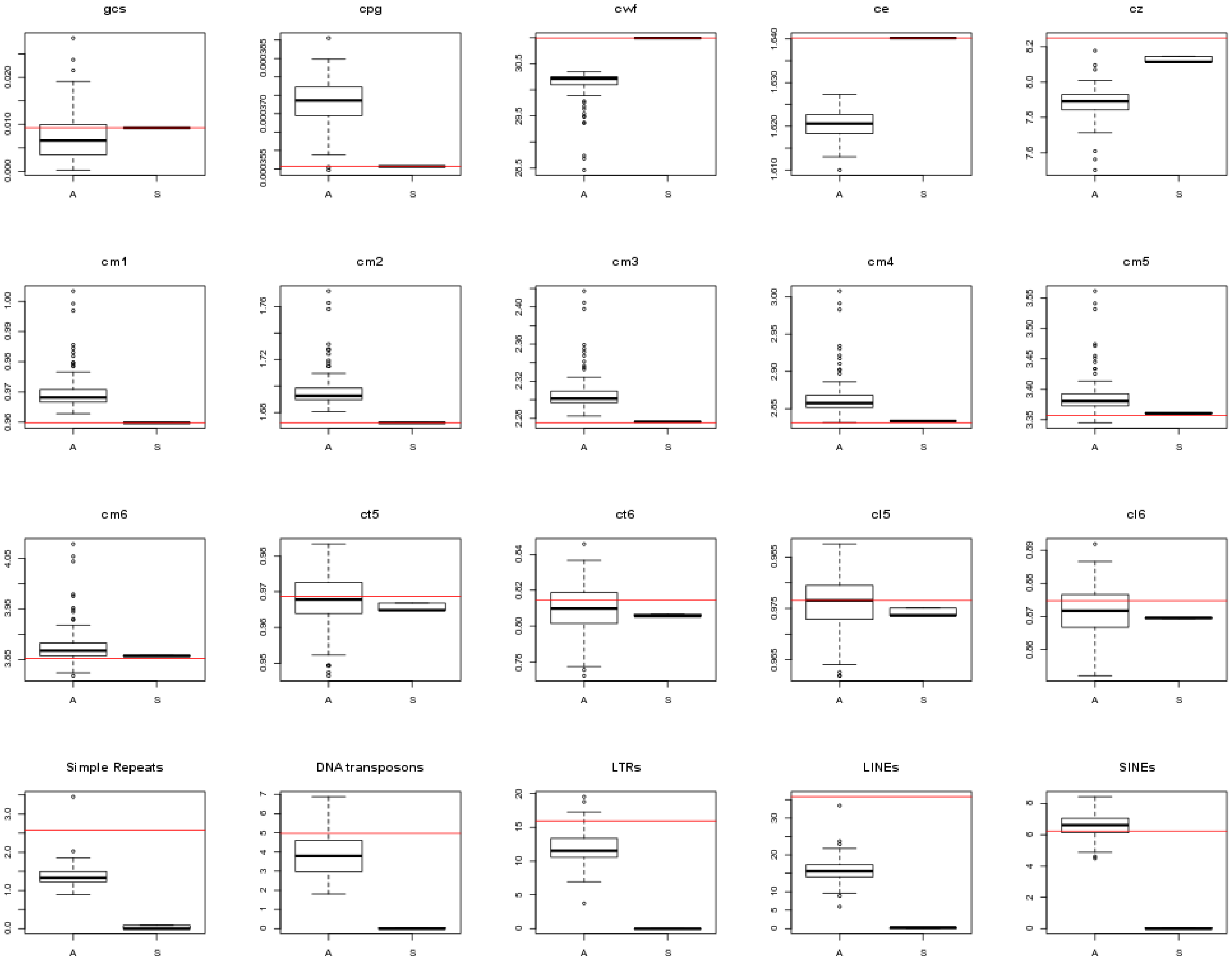

Supplemental Figure 3j. Composition, complexity and repetitive percentage distributions for 100 artificial sequences (A) created after human intergenic region in chr11:24283763-24383763 with similar length, G+C content and fraction of repetitive elements and 100 dimer permutations of the same sequence (S). The red line shows the value in the original intergenic region.

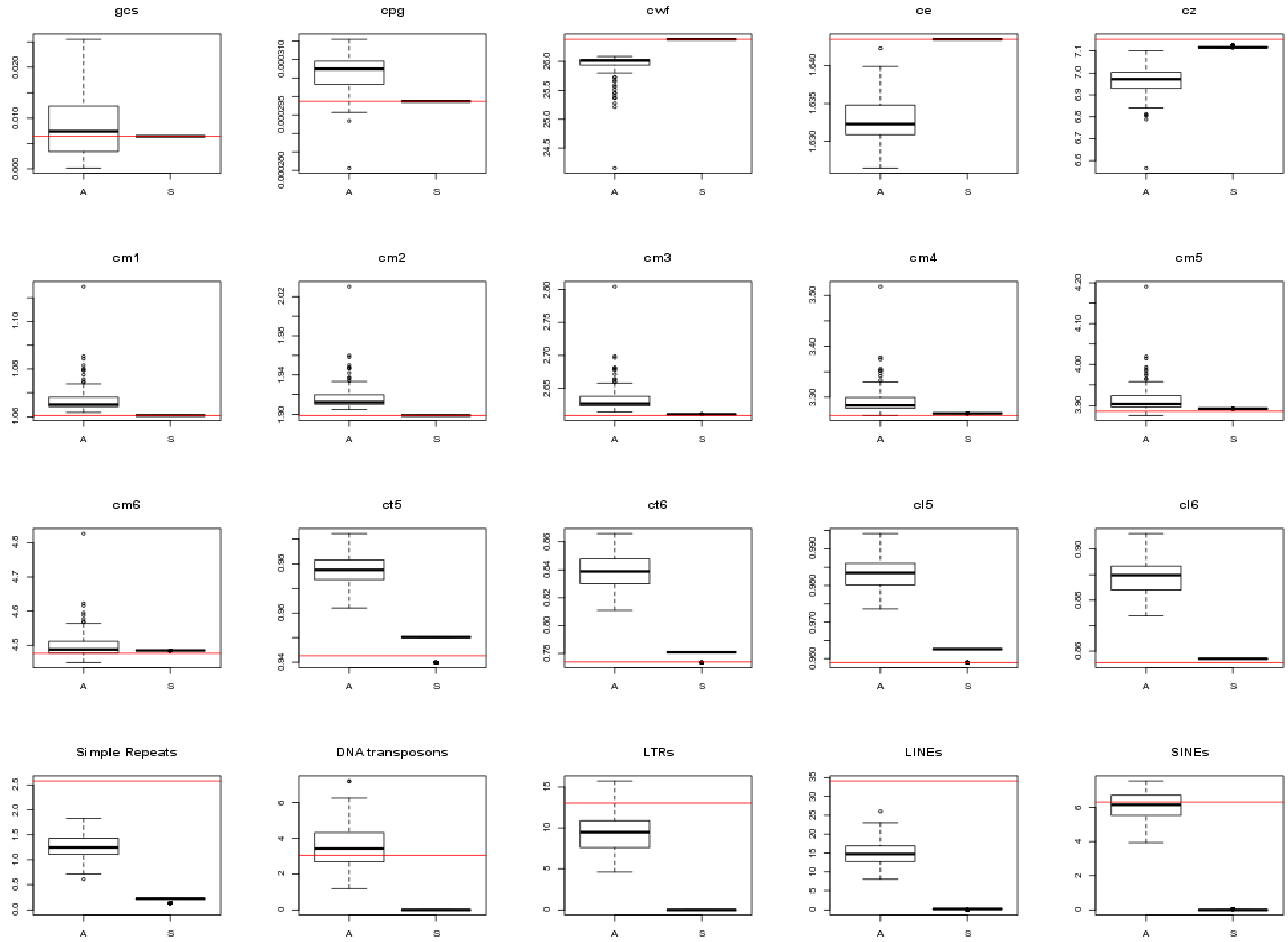

		**hg19 to**													
	hg19	AilMel1	BosTau4	CalJac3	CanFam2	CavPor3	EquCab2	LoxAfr3	Mm9	OryCun2	PanTro3	PonAbe2	RheMac2	Rn4	SusScr2
Intergenic	1,201,929,677	14,057,303	9,218,352	132,165,086	12,077,697	2,443,683	23,872,115	12,371,470	442,637	4,706,197	924,520,058	649,181,306	366,910,309	347,715	8,784,330
Intronic	403,585,028	11,413,214	9,167,830	67,980,442	9,961,215	3,299,607	19,180,879	12,804,354	1,638,538	4,982,644	337,053,195	258,593,890	162,675,529	1,578,369	8,355,726

Supplemental Table 1. Total bases covered in intergenic and intronic regions in all species tested.
